# “When he is around, I’ll take the PrEP, but when he is not, I will not take PrEP”: key influences on PrEP use decisions among women attending family planning clinics in Kenya

**DOI:** 10.3389/fmed.2025.1552132

**Published:** 2025-07-09

**Authors:** Vallery Ogello, Kristin Beima-Sofie, Sandra Urusaro, Mercy Awuor, Annabell Dollah, Winnie Atieno, Cynthia Wandera, Daniel Matemo, Jennifer F. Morton, Kenneth Ngure, John Kinuthia, Kenneth K. Mugwanya

**Affiliations:** ^1^Partners in Health Research and Development, Center for Clinical Research, Kenya Medical Research Institute, Nairobi, Kenya; ^2^Department of Global Health, University of Washington, Seattle, WA, United States; ^3^Research & Programs, Kenyatta National Hospital, Nairobi, Kenya; ^4^School of Public Health, Jomo Kenyatta University of Agriculture and Technology, Nairobi, Kenya; ^5^Department of Epidemiology, University of Washington, Seattle, WA, United States

**Keywords:** HIV prevention, PrEP use decisions, family planning clinics, implementation science, women of childbearing age

## Abstract

**Background:**

Women of childbearing age in sub-Saharan Africa (SSA) face a disproportionately high risk of HIV acquisition. Although oral PrEP has been universally scaled up for individuals at significant risk of HIV, its uptake and sustained use remain suboptimal. Understanding PrEP use decisions offers insights into context-specific barriers and facilitators to its utilization.

**Methods:**

From September to November 2023, we conducted a qualitative study nested in a larger prospective, open-label clinical trial (FP-Plus). We conducted in-depth interviews (IDIs) with younger women (ages 15–24) and older women (ages ≥25) who declined, delayed, discontinued, or restarted PrEP during the study. IDIs were conducted at two FP clinics by trained Kenyan social scientists and were audio recorded, translated, and transcribed. We analyzed data using inductive and deductive thematic analysis through the lens of the theory of planned behavior (TPB) to explore experiences, beliefs, and rationale among women who made various PrEP decisions.

**Results:**

We interviewed 64 women, including 40 younger women and 24 older women, all of whom declined, delayed, discontinued, or restarted PrEP (n = 16 women/category). The median age of these women was 24 years (IQR, 23–30). The majority of participants (86%, 55/64) were using family planning methods, primarily injectables (42%, 23/64). PrEP discontinuation or restart was primarily influenced by changes in HIV risk dynamics. Agency and perceived HIV risk were pivotal factors in PrEP use decisions, shaping participants’ ability to practice effective prevention adherence (TPB: perceived behavioral control). Women who declined PrEP cited a lack of autonomy, partner influence, and insufficient information (TPB: social and subjective norms). Low self-efficacy influenced decisions to delay or decline PrEP (TPB: behavioral beliefs and attitudes). In addition, challenges with PrEP pill size, taste, and texture were perceived as barriers to swallowing pills among all groups of women. Participants expressed a preference for alternative PrEP formulations, such as injectable PrEP, due to perceived ease of use, privacy, and potential to support adherence.

**Conclusion:**

PrEP discontinuation and restart cycles largely reflected changes in HIV risk. Women who decline or delay PrEP may benefit from personalized support to improve their autonomy, recognizing that HIV risk persists during periods of PrEP delay.

## Introduction

Globally, in 2023, women and girls (of all ages) accounted for 44% of all new HIV infections, despite HIV incidence decreasing among other populations (1, 2). Women of childbearing age in sub-Saharan Africa (SSA) face a disproportionately high risk of HIV infection compared to their male peers, with younger women (ages 15–24) being the most affected (3). This disparity stems from physiological differences and socio-cultural factors such as economic inequality, gender-based violence, and limited ability to negotiate condom use (4–6). Oral pre-exposure prophylaxis (PrEP) is safe, effective, and efficacious among people at substantial risk of HIV acquisition (7). In Kenya, oral PrEP has been available since 2016 and scaled up for people at significant risk of HIV, as aligned with the national guidelines (8, 9). However, oral PrEP uptake and persistence remain low among women of childbearing age (10).

In Kenya, PrEP is primarily delivered in HIV clinics. However, barriers such as stigma and privacy concerns in these settings further contribute to low PrEP uptake (11–13). Evidence suggests that delivering PrEP in family planning (FP) clinics is both feasible and preferable (14, 15). The World Health Organization (WHO) recommends the triple integration of services for sexually transmitted infections (STIs), HIV, and pregnancy in high-burden HIV settings (16). Although the Kenya Ministry of Health has provided guidelines for PrEP delivery in reproductive health clinics (17), implementation of PrEP services within family planning clinics remains limited. A recent survey in Kenya assessing knowledge and uptake of PrEP in FP clinics demonstrated high PrEP awareness (89%) and very low uptake of PrEP (only 4%) in FP clinics (18). Understanding the decision-making process for PrEP use within family planning clinics can help address barriers and inform strategies to optimize integrated service delivery and prevent new HIV infections among women.

We conducted a qualitative study nested within the FP-Plus project (15), a programmatic study evaluating the implementation of PrEP delivery in 12 public health FP clinics in western Kenya. Healthcare providers received on-the-job training using the approved case-based interactive Kenya MOH PrEP training curriculum (19). Training included content-specific information on HIV risk assessment, PrEP, FP, STI testing and treatment, and partner testing to facilitate comprehensive service delivery in sexual reproductive health and PrEP. MOH/NASCOP and the Kisumu County Health supervision team provided regular technical support and audits for process indicators of implementation progress. For 2 years, 25,456 women accessed services across 12 FP Plus clinics, 16,989 of whom were screened for HIV risk and PrEP eligibility. We explored key influences on PrEP use decision-making among a subset of women of childbearing age attending two study sites who decline, delay, discontinue, or restart PrEP. Understanding this information is crucial in ensuring health interventions are designed to address context-specific barriers and promote effective use of oral PrEP.

## Methods

### Study design and setting

This qualitative study was part of FP-Plus, a stepped-wedge randomized clinical trial aimed at integrating HIV prevention and PrEP care services for women in family planning clinics in Kisumu, Kenya (15). HIV prevalence in Kisumu is 3.4 times higher than the national prevalence of 19.9%, and women exhibit a prevalence of up to 28% (20, 21).

### Study population

Participants included reproductive-aged women (≥15 years) who accessed services at two of 12 FP-Plus study clinics and were HIV-negative at the time of recruitment. The two study clinics were purposively selected to reflect diversity in service delivery and context and allow an in-depth understanding of women’s experiences. We used stratified purposive sampling to ensure diverse representation by age and PrEP use. We purposively selected women aged 15–24 and older women of reproductive age (≥25 years of age) and stratified recruitment to include women making different PrEP use decisions, including those who (1) declined PrEP defined as being eligible for PrEP as per the Kenya risk assessment tool (RAST) and failed to take it up, (2) delayed initiation defined as postponement to take PrEP after successful HIV risk screening, (3) discontinued use defined as stopping to take PrEP after initiation, or (4) restarted PrEP defined as beginning to use PrEP again after a period of discontinuation of more than 2 weeks. Participants were recruited by facility staff during routine clinic visits, and interested participants were referred to the study staff for scheduling.

### Data collection

We conducted in-depth interviews between September and November of 2023. Our sample size was guided by a qualitative content analysis approach, which focuses on attaining information power (22). Based on the research question, we anticipated that conducting 16 interviews per category would provide sufficient information, resulting in a total sample size of 64 interviews. Interviews were conducted using piloted, semi-structured interview guides informed by the TPB to understand key influences on PrEP decision-making. The semi-structured guides included experiences accessing HIV prevention and family planning services, key decisions related to initiating, delaying, declining, or restarting PrEP, the potential impact of longer-acting formulations on PrEP use decisions, and participants’ knowledge and beliefs about HIV prevention. Interviews were conducted by experienced bachelor-level female social scientists (MA, AD, and WA) in the participants’ preferred language (English, Swahili, or Dholuo). All three social scientists are Kenyan and have significant experience interacting with women in the field of HIV research. The interviewers had a non-clinical background, with no direct role in the implementation of the study or delivery of PrEP services to the women. The interviewer’s external position and gender may have likely enhanced reflexivity, contributing to rich, contextualized accounts that may be transferable to similar health system contexts. Demographic data were collected prior to interviews via REDCap, a web-based application hosted at the University of Washington (23). Participants received KSH 600 (~5 USD) as compensation for their time participating in study activities. Interviews were audio recorded with permission, transcribed, and translated into English as needed. Transcripts underwent quality checks before analysis. Interviewers wrote targeted debrief reports following each interview to capture key concepts in real-time.

### Data analysis

We used a thematic analysis approach using a combination of inductive and deductive methods. An initial codebook was developed inductively by SU and KBS based on open coding of a subset of transcripts and review of targeted debrief reports. The codebook was further refined through review of additional transcripts by the larger coding team (KBS, SU, WA, HL, MA, AD, and VO) to arrive at a final version of the codebook. All transcripts were coded independently by one member of the team using the final version of the codebook, and a subset of transcripts was reviewed by another member of the team to note code agreement and discrepancies. All coding discrepancies were resolved through group discussion among the coding team members. Codes and emerging concepts of importance were then deductively compared to domains within the TPB (24) and applied to transcripts by WA, MA, AD, and VO. The TPB suggests that human social behavior follows a more or less formulated plan and may be applied in health interventions to describe “decision support” (24, 25). The team independently coded transcripts to generate themes related to decisions regarding PrEP use, including: (1) perceived behavioral control, (2) social and subjective norms, and (3) behavioral beliefs and attitudes. Weekly debriefs resolved coding discrepancies in the TBP code application. Coding was supported by ATLAS.ti (Scientific Software Development GmbH, Berlin, Germany) http://atlasti.com. After coding was completed, we generated thematic summaries from the coded excerpts and identified emerging patterns and theoretical ideas from the data to refine the emerging themes. Our qualitative methods adhered to standards for reporting qualitative research (SRQR) (26).

### Human subjects considerations

The FP-Plus study was approved by the University of Washington Human Subjects Review Committee and the Kenyatta National Hospital-University of Nairobi Ethical Review Committee. All participants provided written informed consent for qualitative interviews.

## Results

A total of 64 women, namely 40 adolescent girls and young women (AGYW) and 24 older women of reproductive age, completed in-depth interviews. Women were stratified to include an even number of women who chose to decline, delay, discontinue, or restart PrEP (*n* = 16/category, including 10 AGYW and 6 older women). Among all participants, the median age was 24 years (IQR: 23–30). Approximately half of them had completed a high school level of education (39%, 25/64) and were in a monogamous marriage (48%, 31/64). The majority of participants (86%, 55/64) were using family planning methods, primarily injectables (42%, 23/64). A few of them (14%, 9/64) were not on any family planning method at the time of the interview due to unknown reasons (Table 1).

**Table 1:** Social demographic characteristics of IDI participants in the FP-Plus Study.

Variable	*N* (%) or median (IQR)
Age in years	24 (23–30)
Highest level of education completed	
Diploma level	3 (5%)
Polytechnic/Diploma	2 (3%)
Primary school level	20 (31%)
Secondary school level	25 (39%)
University/college	14 (22%)
Marital status	
Married monogamous	31 (48%)
Married polygamous	6 (9%)
Never married/single	18 (28%)
Separated /divorced	7 (11%)
Widowed	2 (3%)
Number of sexual partners	
None	2 (3%)
One	51 (80%)
Two	7 (11%)
Three	3 (5%)
Four	1 (1%)
Partner has HIV	
Yes	3 (5%)
No	43 (67%)
Do not know	18 (28%)
Initiated PrEP	
No	17 (27%)
Yes	47 (73%)
Currently on a family planning method	
No	9 (14%)
Yes	55 (86%)
Types of family planning (*n* = 55)	
Injectable	23 (42%)
IUD	3 (5%)
Implant	19 (35%)
OCP	7 (13%)
Other^a^	2 (4%)

SD, standard deviation; IQR, interquartile range.

a Emergency contraception.

**Figure 1:**
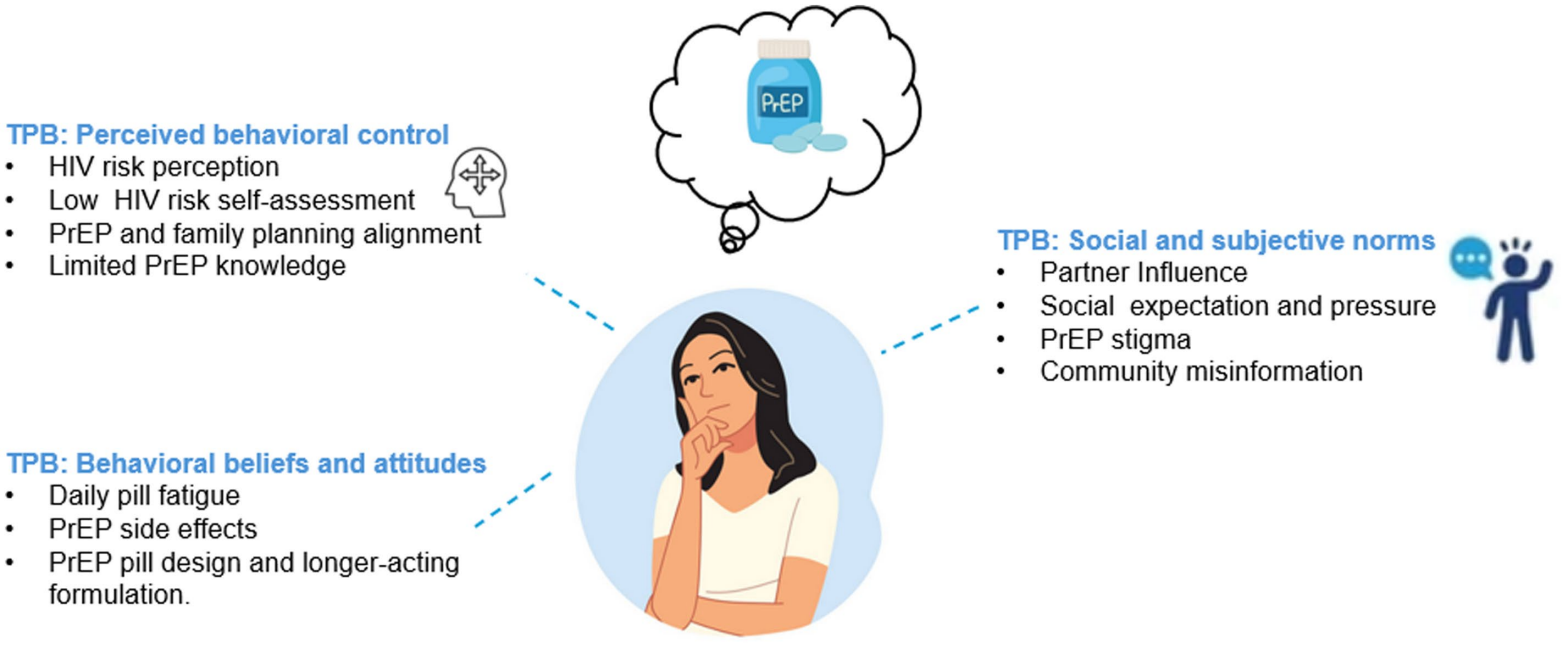
Key influences on PrEP use decision among women attending family planning clinics in Kenya.

Perspectives on decisions regarding PrEP use among women are influenced by a complex interplay of enablers and obstacles. While HIV risk dynamics and access to information are important PrEP use enablers, low decision-making power, partner influence, PrEP stigma, and inadequate information considerably impede PrEP use. Overall, participants described how social situations and PrEP knowledge acted as primary drivers for their decisions to use or not use oral PrEP, which was further compounded by their perceived agency to enact choices. Key reasons for PrEP discontinuation and restart were perceived changes in HIV risk, which influenced women’s motivation and subsequent adherence. For example, women reported using PrEP only during seasons of increased HIV risk, such as when partners might be in town (TPB: perceived behavioral control). Participants who declined PrEP were heavily influenced by a perceived lack of autonomy in decision-making, partner influence, and lack of accurate information about PrEP (TPB: social and subjective norms). Additionally, other key influences on PrEP delay, decline, and discontinuation included challenges of PrEP pill design, as it was perceived as ‘difficult to swallow’, as well as the perceived low self-efficacy in their ability to adhere to daily medication (TPB: behavioral beliefs and attitudes). All participants were interested in alternative PrEP modalities, such as long-acting injectable PrEP, which were perceived as private and easy to adhere to (TPB: behavioral beliefs and attitudes) (Figure 1). Additional details of key influences on decision-making, aligned with the TPB, are described below.

### Perceived behavioral control

A primary driver of making decisions regarding PrEP use was HIV risk perception. Although the clinical risk RAST placed them at higher HIV risk, participants who declined PrEP believed that they were not at high risk based on their assessment of sexual behavior.

“Okay, when I came for FP generally, I was asked if I am willing to take PrEP. But to me, I did not see it as meaningful to me because of that exposure thing…I was feeling I am not at risk of getting HIV [29-year-old, Declined].

Participants’ low-risk assessment was based on their participation in routine HIV testing at the clinic, their condom use, their partner travel patterns, and being in monogamous relationships. This same low-risk perception also led many participants who had initiated PrEP to discontinue its use. Importantly, personal risk assessment facilitated PrEP restart among participants when the perceived risk factors changed and HIV risk increased. Risk perception interacted with the perceived agency to influence prevention-effective adherence, whereby participants only used PrEP when they needed it. For this reason, some participants restarted PrEP after a period of discontinuation, highlighting that PrEP use aligned with seasons of risk.

“When my husband is around, I’ll always take it [PrEP], or when I know that he’ll be coming. So, maybe I’ll just take that PrEP and at times maybe he is out of the country, so, I do not see the need to take that PrEP because I only have one partner. Yeah, so when he is around, I’ll take the PrEP, but when he is out of the country, I will not take the PrEP” [27-year-old, Restart].

One woman who discontinued PrEP earlier reported restarting its use after a positive pregnancy test and the need for HIV prevention during these maternal periods. Other women who restarted PrEP reported changes in other higher risk behaviors, such as increased alcohol use, that prompted their decisions to restart PrEP.

“What motivated me is the fact that I usually drink alcohol and get drunk, so that day I was drunk, then I remembered that I had not taken PrEP and where I was, it was a risky situation” [34-year-old, Restart].

Perceived HIV risk also influenced the participants’ decisions to delay PrEP use. Participants who delayed PrEP use reported their limited knowledge about PrEP as a key factor influencing their ability to make informed risk assessment and PrEP initiation decisions. Many reported feeling more confident in their ability to assess their personal HIV risk by their next visit, following the initial PrEP offer and decided to initiate PrEP based on an accurate assessment of identified risk factors.

“I started having an affair and so I decided to take it to protect myself and him.” [30-year-old, Delayed].

Participants also described how alignment, or lack of alignment, between oral PrEP and contraceptive use modalities influenced PrEP use decisions. For some participants using contraceptive pills, the idea of taking another daily oral pill was prohibitive of PrEP initiation due to perceived adherence challenges associated with having to consistently take two medications [PrEP and contraceptive pills].

“I take pills [contraceptives] every day and then again PrEP, so I said no, PrEP decline [29-year-old, Declined].

Of note, participants described being more comfortable using contraceptive pills when compared to oral PrEP, because they were ‘smaller.’

“The tablets [PrEP] also are too big [Laughs]. I only agreed to pills [contraceptives] because they are small” [24-year-old, Declined].

Although some women expressed initial hesitancy about PrEP use due to concerns about taking two medications, their continued use of oral contraceptives reinforced their confidence in their ability to adhere to a daily oral medication. Adherence to oral contraceptives facilitated belief in their ability to effectively adhere to oral PrEP.

“What motivated me is that I used to find it so hard to take any medication, even the ones for just treating illness were so hard for me to take, but the more I continued to take daily pills [contraceptives], it convinced me that even this one for PrEP I can take. The same way I take pills [contraceptives] every day is the same way I can take it, so that my life can be safe” [23-year-old, Delayed].

### Social and subjective norms

Partners’ opinions significantly influenced PrEP decisions. Some women reported delayed PrEP use due to fears about partners’ reactions. Participants highlighted how a lack of privacy in their houses was a challenge to PrEP use for women, where disclosure was a concern.

“I will not take PrEP because another thing is that it needs consent from the partner what if I took PrEP here and my partner came and found those drugs, he would not know it is PrEP he would say I’m taking drugs, but I do not want him to know, so that’s also another fear, and the way they look like ARVs” [laughs] [29-year-old, Declined].

Some participants who initiated PrEP reported discontinuing it after their partners found PrEP in the house. In some instances, partners either threw away the pills or forced their partners to stop taking PrEP. Women believed that their partners acted this way because they did not have accurate information about PrEP and perceived PrEP pills as being ARVs.

“They [PrEP] are pills that even if someone gets in your house, explaining that they are not ARVs is difficult because of the size and how they look.” [24-year-old, Declined].

In addition to concerns about partners, fear of experiencing stigma from disclosing PrEP use to others was also a key decision factor leading to declining it. Participants were particularly concerned about what others in the community might say if they discovered their PrEP use. Participants felt that a lack of information among community and family members about PrEP would lead others to perceive them as having irresponsible sexual behaviors or believe they were living with HIV. Part of this confusion stemmed from beliefs that oral PrEP was used only by individuals in sero-different relationships or people with multiple sexual partners. Notably, healthcare providers also influenced PrEP initiation. The continued delivery of PrEP information during routine clinic visits corrected misconceptions among participants and equipped participants with more confidence to start PrEP after an initial delay.

“When I came [clinic visit], they were telling me the importance of taking those drugs [PrEP] and that is why I decided again … when they were explaining I understood if this person [partner] is far and I’m here and I want to stay safe, at least I need to protect myself before somebody else protects me. So that’s when I decided, and I started taking them. [28-year-old, Delayed].

Social expectations and social pressures around joint decision-making also influenced PrEP use decisions. Some participants felt that support or approval from their peers or partners was essential before initiating PrEP. Some women reported that, despite obtaining information from a healthcare provider, they wanted a second opinion from someone ‘trusted’. In this case, women sought ‘approval’ from people who had experience using PrEP or from their partners. AGYW were more likely to seek a second opinion compared to older women of childbearing age.

“They [providers] told me yes, but I had some doubts, I wanted to hear more from people that I know. I did not want to hurry and use something that might harm me.” [23-year-old, Delayed].

Some women also reported not being ‘ready’, choosing to delay PrEP initiation to have more time to make a decision. These women described not being psychologically prepared to take PrEP when initially counseled about it during their routine FP visit. After allowing time for deliberation and self-assessment, these participants initiated PrEP at a later visit when they felt mentally prepared.

“Yes, I delayed, again, when I was coming, I was not ready for that. I was not prepared that they were going to give me PrEP. I knew I was coming for family planning” [24-year-old, Delayed].

### Behavioral beliefs and attitudes

Participants reported potential and actual challenges with their daily use of oral medication that influenced their discontinuation or decline. A young woman who declined PrEP reported that she was initially excited about PrEP, but the dosing frequency was a ‘turn off’. Participants who declined PrEP were aware that they would struggle to adhere to a daily medication. Pill fatigue facilitated taking a ‘drug holiday’ among participants who discontinued PrEP as they cited, “I was tired,” or “I wanted to rest.” Some participants based their adherence concerns on what they had heard from peers, noting shared beliefs in struggling to maintain daily adherence.

“Okay, there is another friend of mine who was using PrEP and she told me taking PrEP is very difficult. It is something you will take daily like those who are on HIV treatment. So, you will take it as long as you are at risk of getting HIV. So, I felt that I could not take medication daily. So, when I was asked if I could take it, I just said outrightly, No!” [20-year-old, Declined].

Given the challenges of taking a daily oral pill, participants preferred access to other long-acting PrEP formulations, such as monthly oral, injectable, or implantable PrEP, highlighting that these alternative formulations would be easier to use, reduce the burden of daily medication use, and ensure privacy and confidentiality.

“I just take the pill because the injection is not yet available, but I would switch to it because it is longer lasting, and I do not have to take it every day” [23-year-old, Discontinued].

Although excited about the longer-acting PrEP formulations, participants also noted concerns about the potential irreversibility of side effects or potential contraindications with family planning (i.e., getting a PrEP injection at the same time as a contraceptive injection at an FP visit).

“But the implant also is a no! because maybe they want to put it in the arm and Jadelle[contraceptive] is also in the arm, so two things cannot be in your arm running at the same time” [30-year-old, Delayed].

Participants who declined or delayed PrEP initiation reported not having clearly understood the information given by providers, including information about how to take PrEP and how it works. Additionally, existing misinformation about side effects, such as PrEP causing body rashes, jaundice, foot swelling, infertility, and reduced libido, caused women to delay or decline PrEP.

“I have seen people taking PrEP suffer from jaundice, their eyes turn yellow, vomiting, fatigue, and nausea, and then mostly the people who take PrEP, not all but when they come with the card for retesting before even, he or she gives you the card you can suspect they are on care [living with HIV]. I do not know why. You just see someone has sunken eyes and I do not know if it [is just] me that has these beliefs.” [24-year-old, Declined].

Importantly, participants felt that, when given comprehensive information and the opportunity to correct misinformation, they were more comfortable with taking PrEP. For instance, a young woman reported that the more she frequented the family planning clinic, the more she learned about PrEP, leading her to initiate PrEP after a few information sessions.

“So, the more they talked to me, the more I accepted. They educated me on the importance and how it can help me, so I decided to use it.” [22-year-old, Delayed].

## Discussion

Our findings highlight the complex interplay of enablers and obstacles to PrEP use as they relate to behavioral beliefs, perceived risk, and social influences. In addition to low uptake of oral PrEP, the need to scale up HIV prevention efforts among women of childbearing age has been described elsewhere (27). Our findings indicate that the successful uptake and sustained use of PrEP among women necessitate a multi-sectoral approach that should extend beyond individual-level interventions. Dynamic decision factors highlight how women make decisions when choosing to use PrEP. We contribute to the growing body of knowledge on real-world factors that influence the uptake and persistence of PrEP for women, with the most salient being, among others, family and partnership dynamics (28). Uniquely, we present precursors of motivation to perform a behavior (PrEP use), including HIV risk dynamics, individual factors, product-specific factors, and social and subjective factors.

Low decision-making power and perceived low self-efficacy were key reasons for PrEP delay among women, especially AGYW. Some participants reported reduced agency in decision-making, noting a desire to obtain approval from their peers or partners before initiating PrEP. For other women, the delay was related to limited knowledge on PrEP. A meta-analysis study conducted in 23 SSA countries found that low decision-making power was characterized by a lack of knowledge and low socio-economic status (29). Borrowing lessons from contraceptive uptake, modern contraception uptake has been shown to improve with knowledge and joint decision-making (30, 31). Similarly, our study demonstrated that regular clinic visits contributed to improved knowledge, which facilitated PrEP initiation following initial PrEP postponement. However, the contexts of joint decision-making may differ given the circumstances of pregnancy prevention versus HIV prevention, and young women may benefit from strategies that improve autonomy.

Perceived low self-efficacy and adherence challenges caused PrEP discontinuation and delays. Women in our study described delayed PrEP due to a ‘lack of psychological preparedness’ at their routine FP visit. PrEP postponement may be appropriate in supporting women’s abilities to make informed decisions and leading to improved adherence later on. A large PrEP program in Namibia found that AGYW who delayed starting PrEP were 2.89 times more likely to persist with the medication over time (32). However, timely initiation is still crucial due to the continued risk of exposure during periods of delay (33). PrEP adherence remains challenging, especially among young women who initiate PrEP. For instance, a prospective cohort study among young women in Kenya recorded suboptimal adherence, even with continued program attendance (34). The effectiveness of PrEP is user-dependent, and other long-acting PrEP formulations may address barriers to adherence and persistence, such as privacy and stigma concerns (35).

HIV risk dynamics played a crucial role in PrEP use restart and discontinuation. Women were cognizant of their HIV risk factors, such as alcohol use, multiple sexual partners, or mistrust within sexual relationships, which influenced them to reinitiate or stop PrEP. Women also declined PrEP due to perceived low HIV risk. A study conducted in South Africa and Zimbabwe found a correlation between PrEP use and HIV risk, which demonstrated prevention-effective adherence (36). However, women still struggle with inaccurately assessing their HIV risk, and PrEP adherence has been shown to misalign with HIV risk among Kenyan women (37). Aligning PrEP and FP may influence PrEP uptake across all formats, as concerns about pill burden and potential contraindications associated with alternative formats, such as injectables, can affect perceptions and decisions. Drawing insights from family planning, providing PrEP alternatives that would meet individual needs and circumstances would support women in making PrEP-use decisions (38). Additionally, multipurpose HIV prevention technologies (MPTs), such as the dual-purpose pill (DPP), offer a cost-effective approach to prevention by addressing both HIV and pregnancy concerns. These products may be appealing to women since they have the potential to effectively reduce the burden of using multiple interventions and provide universal access to protective measures (39, 40). Future research should explore the interactions between contraceptive use and various PrEP formulations. This exploration could provide valuable insights into how combined contraceptive and PrEP strategies affect adherence, efficacy, and overall sexual health outcomes. Understanding these dynamics will be crucial for developing integrated approaches to prevention in reproductive health clinics.

This programmatic study has important strengths and limitations. The key strength is the use of in-depth interviews that provided meaning to decision-making regarding PrEP use among women within the real-world setting and our strategic sampling of women who made different decisions when considering PrEP uptake (decline, delay, discontinue, restart). The use of TPB also provides a comprehensive framework of PrEP use behavior influenced by perceived behavioral control, attitudes, and social norms, which may guide behavior-informed interventions. Comparing perspectives between these groups allows the characterization of factors influencing initial PrEP use as well as PrEP persistence. The primary limitation is that women were offered PrEP while already accessing FP care services, and their perceptions may differ from women not accessing any clinical services. We only report findings among women accessing FP clinics, which may be different in other PrEP priority populations. Despite these limitations, understanding PrEP use decisions among women of reproductive age can inform holistic approaches to ensure access and uptake of HIV prevention among this priority population.

## Conclusion

We describe PrEP use decisions framed in the context of the theory of planned behavior to understand how multiple factors and context-specific challenges combine to influence PrEP decision-making among women of childbearing age. Our study highlighted how women’s decision-making process surrounding the continuation, discontinuation, restart, decline, or delay of PrEP at family planning clinics is multifaceted and influenced by individual and social factors. We found that PrEP discontinuation and restart cycles were mostly appropriate as they are related to changes in HIV risk. PrEP decline and delay are substantially influenced by low decision-making power and limited knowledge. To empower women to make informed decisions, it is essential to provide personalized support to improve their autonomy, particularly those who delay or decline PrEP, given that seasons of HIV risk persist during delay periods.

## Data Availability

Anonymized data will be available upon author request and under appropriate data-sharing agreements to those who provide a methodologically sound proposal. Requests to access the datasets should be directed to International Clinical Research Center at the University of Washington (icrc@uw.edu).
