# Supplementary material for: “When he is around, I’ll take the PrEP, but when he is not, I will not take PrEP”: key influences on PrEP use decisions among women attending family planning clinics in Kenya
Source: Front Med (Lausanne). 2025 Jul 9;12:1552132. doi: 10.3389/fmed.2025.1552132 (PMC12283639; doi:10.3389/fmed.2025.1552132)
Supplement: Supplementary file 1 [file Data_Sheet_1.pdf]

FP Plus - IDI Question Guide
Women

Interviewer Instructions:
The following is a guide. Try to ask all the questions below in the order given, but it is more important to maintain the flow of discussion. Suggested probes have been included. Start with the following introductory script:

****
Before turning on the recorder, start with the following introductory script:

Hi, my name is [interviewer name]. Thank you for agreeing to participate in an interview today.

The goal of this research is to find out more about how best to reach women for HIV prevention services including understanding facilitators and barriers to implementing HIV prevention services in family planning clinics in Kenya.

We're interested in your thoughts about providing PrEP in family planning clinics.

During our discussion today, I will ask you questions that you are free to answer in any way you wish. Feel free to elaborate on any of your points. If a question is unclear to you, please feel free to ask me to explain it.

I would like to record the discussion so I don't miss anything that you say. I will not include your full names on any documents or in the recording. Your answers will be kept confidential, which means we will keep what you say private from others. Is it okay if I record our discussion? [Wait for the participant to give verbal consent to recording]

Before we start I would like to remind you that there are no wrong answers during our discussion. We are interested in knowing what you think, so please feel free to be open and share your point of view. We hope you can help us understand your experiences getting HIV services within FP clinics so that we can improve provision of these services in the future, Your comments about what worked well are just as helpful as your comments about didn't work well.

If you want to stop the discussion at any time, just let me know. Do you have any questions for me before we get started? [Wait for participant to respond – answer any questions they have]

I am turning on the recorder now.

****
Before beginning interview questions, please read the following script for the recording:

Today is [day of week], [month, day, year] and it is now [time of day]. This is interview [ID number] conducted at [discussion location].

You are now ready to begin asking the questions outlined below.

1. First, can you tell me a little about yourself and your experiences getting health services?
    - <u>What positively influences your decisions to come to the clinic for services?</u>
    - <u>What negatively influences your decisions to come to the clinic for services?</u>
    - ***Possible probes:*** quality of services, wait times, provider attitudes, provider knowledge, likes and dislikes, positive or negative past experiences, beliefs or experiences of peers or family, beliefs about the healthcare system generally, challenges accessing services

2. Can you tell me about your experiences accessing FP services and using FP?
    - What methods have you used before? Why pick that/those one(s)?
    - What method, if any, do you currently use?
    - How does your relationship with your partner influence your FP method choice?
    - How do experiences with FP of peers or family influence your decisions to use FP methods?
    - What types of things at the clinic influence your FP method choices?
    - ***Possible probes:*** methods used, reasons for using FP, rationale (knowledge/beliefs) for method selection, positive/negative experiences at the clinic, previous or ongoing positive/negative experiences with use (side effects, partner reactions, etc), relationships with partners (IPV), family, or providers (attitudes, trust, communication), community norms, logistics/cost (wait time, concern about loss of work, travel time), stigma, others?

3. Can you tell me about your experiences accessing and using HIV prevention services, such as HIV testing or condoms?
    - What HIV prevention services or strategies have you used before?
    - What motivates you to get HIV testing? What prevents you from getting HIV tested?
    - What motivates you to use condoms? What prevents you from using condoms?
    - ***Possible probes:*** HIV prevention knowledge and beliefs, personal experiences testing (including self-testing and partner testing), provider attitudes, rationale for accessing/using services/methods, barriers to accessing services, barriers and facilitators to acceptability, feasibility and uptake, role of partners, family, community in decision-making

4. During your time in the study, you were offered the opportunity to take PrEP. What was your experience like being offered PrEP while attending the FP clinic?
    - <u>*All*</u> - What influenced your initial decision to take or not take PrEP?
        1. Did your current family planning method choice affect your PrEP decision? How?
    - <u>*Delayed initiation*</u> – Why did you wait to initiate PrEP? How long did you wait? What were the main influences on your decision?
        1. Think back to the last time you took a PrEP pill, what motivated you to take your pill?
        2. We want to know more about how people take PrEP so that we can provider better counselring for women about PrEP. Did you take PrEP pills every day, or following some other strategy? If you didn't take them every day, what informed how you chose to take your pills?
        3. Think back to the last time you had sex – did you use PrEP around this time? Why or why not?
    - <u>*Discontinued PrEP*</u> – How soon after starting to take PrEP pills did you stop taking pills? What influenced your decision to discontinue?
        1. Think back to the last time you took a PrEP pill, what motivated you to take your pill?
        2. We want to know more about how people take PrEP so that we can provider better counselring for women about PrEP. Did you take PrEP pills every day, or following some other strategy? If you didn't take them every day, what informed how you chose to take your pills?
        3. Think back to the last time you had sex – did you use PrEP around this time? Why or why not?
    - <u>*PrEP Persisters*</u> – How long have you been taking PrEP? What has helped you persist with taking PrEP?
        1. Think back to the last time you took a PrEP pill, what motivated you to take your pill?
        2. We want to know more about how people take PrEP so that we can provider better counselring for women about PrEP. Did you take PrEP pills every day, or following some other strategy? If you didn't take them every day, what informed how you chose to take your pills?

3. Think back to the last time you had sex – did you use PrEP around this time? Why or why not?

- *<u>All</u>* – If you had to make the decision today about taking PrEP, would you make a different decision? Why?
- *<u>All</u>* – If PrEP has been available in another format, such as an injection, implant, longer lasting/monthly pill, would that have influenced your decision to take PrEP?
- ***Possible probes:*** HIV prevention knowledge and beliefs, perceived/actual HIV risk, fear of HIV, STI risk/diagnosis, PrEP-product (side effects, pill burden), relationships with partners (IPV), family, or providers (attitudes, trust, communication), community norms, logistics/cost (wait time, concern about loss of work, travel time), stigma, others?

5. If we were to continue to offer PrEP and HIV prevention services at the FP clinic, what would you think about that?
    - ***Possible probes:*** What are some of the specific challenges you experience while accessing PrEP? long term barriers and facilitators to acceptability, feasibility and uptake, rationale for decisions to use/not use services now and in the future, considerations for providers in sustaining integrated services, considerations for policy-makers in sustaining integrated services

As we finish taking today, are there any other questions we should be asking people like you to help us understand whether and how PrEP can be integrated into FP clinics?

Is there anything about PrEP, HIV or FP services that you think is important to tell me, but I didn't ask about?

That is all of the questions I have for you today. Thank you for your time. If you do not have any further questions or comments, I will now turn off the recorder.

**1**	Sex: ☐ Female
2	Age:|__|__|years<br><br>**1a** Date of birth: |__|__|__|__|__|__|__|__|
3	What is your highest level of education? *(Highest level of education **completed**)*<br><br>☐none ☐ primary: class _____ (1-8) ☐ secondary: form ______(1-4)<br><br>☐ polytechnic: year____ (1-4) ☐ university/college year _____ (1-6)
4	What is the participant's primary job/occupation?<br><br>☐Unemployed ☐ Student ☐ Employed with steady salary/formal sector<br><br>☐Employed without steady salary/formal sector ☐ Housewife<br><br>☐ Household help<br><br>☐ Other, specify: ____________________________
5	What is the participant's marital status:<br><br>☐Never married ☐ Cohabiting ☐ Married monogamous<br><br>☐Married polygamous ☐ Separated /divorced<br><br>☐Widowed
6	Is the participant in discordant relationship Yes ☐ No ☐
7.	How many sexual partners do you have currently?
8	7a. Participant is at risk of HIV infection as defined by NASCOP: Yes ☐ No ☐

	7b. Was the participant initiated on PrEP Yes ☐ No ☐<br><br>7c. Is the participant still on PrEP Yes ☐ No ☐
Comments:	
Staff initials: Date:	

Date of Interview: |__|__|__|__|__|__|__|__|__|

Interview start time: __ __:__ __ ❑ am ❑ pm

Interview site: ____

Name of Interviewer: ____

Name(s) of other study staff present: ____________________________
